# Calculation of Core-Excited and Core-Ionized States Using Variational Quantum Deflation Method and Applications to Photocatalyst Modeling

**DOI:** 10.1021/acsomega.2c01053

**Published:** 2022-03-16

**Authors:** Soichi Shirai, Takahiro Horiba, Hirotoshi Hirai

**Affiliations:** Toyota Central R&D Labs., Inc., Nagakute, Aichi 480-1192, Japan

## Abstract

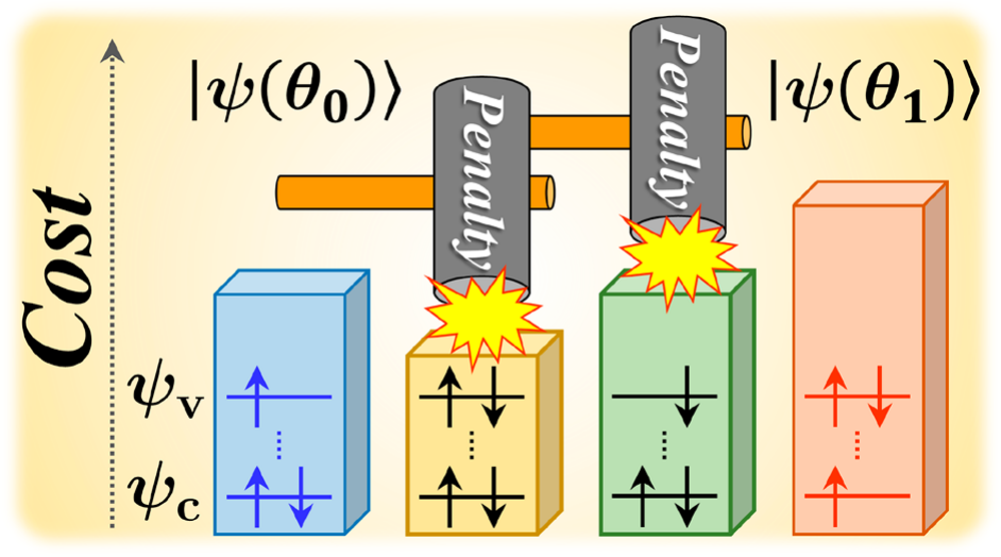

The possibility of performing quantum-chemical calculations using quantum computers has attracted much interest. Variational quantum deflation (VQD) is a quantum-classical hybrid algorithm for the calculation of excited states with noisy intermediate-scale quantum devices. Although the validity of this method has been demonstrated, there have been few practical applications, primarily because of the uncertain effect of calculation conditions on the results. In the present study, calculations of the core-excited and core-ionized states for common molecules based on the VQD method were examined using a classical computer, focusing on the effects of the weighting coefficients applied in the penalty terms of the cost function. Adopting a simplified procedure for estimating the weighting coefficients based on molecular orbital levels allowed these core-level states to be successfully calculated. The O 1s core-ionized state for a water molecule was calculated with various weighting coefficients, and the resulting ansatz states were systematically examined. The application of this technique to functional materials was demonstrated by calculating the core-level states for titanium dioxide (TiO_2_) and nitrogen-doped TiO_2_ models. The results demonstrate that VQD calculations employing an appropriate cost function can be applied to the analysis of functional materials in conjunction with an experimental approach.

## 1 Introduction

The concept of performing quantum-chemical calculations using quantum computers has attracted significant attention.^1,2^ In this regard, the configuration interaction (CI) framework is the most common means of assessing electron correlation. In this framework, the wave function is described as a linear combination of Slater determinants or spin-adapted configuration state functions that represent the electron configurations.^3^ If all of the electron configurations generated from combinations of all the electrons and orbitals of the system are included, the method is termed full CI.^3,4^ Although exact wave functions within an adopted basis set can be obtained using full CI calculations, the computational cost increases quite rapidly as the number of orbitals and electrons increases and can easily outgrow the capacity of standard computers. In contrast, quantum computers could potentially perform the required calculations in polynomial time using the quantum-phase estimation (QPE) algorithm.^5−8^ Unfortunately, the QPE method requires fault tolerance and cannot be practically executed on current quantum computers (so-called noisy intermediate-scale quantum (NISQ) devices) that operate on the basis of qubits without error correction.^9−11^ Accordingly, a quantum-classical hybrid algorithm (termed the variational quantum eigensolver (VQE)) has emerged as a means of allowing quantum-chemical calculations to be performed using NISQ devices.^12,13^

Following the development of the VQE method to compute the ground state for a given system, VQE-based algorithms for excited states have also been proposed, including quantum subspace expansion (QSE),^14^ multiscale-contracted VQE (MCVQE),^15^ subspace-search VQE (SSVQE),^16^ quantum equation of motion VQE (qEOM-VQE),^17^ and others. The variational quantum deflation (VQD) method introduced by Higgott et al. is one such algorithm and allows computation of excited states based on the ground state obtained using the VQE method.^18^ The superior efficiency and accuracy of the VQD approach compared with the SSVQE and MCVQE methods have been demonstrated based on calculations of the excited states for lithium hydride, diazene, and azobenzene.^19^ The VQD method has also been used to investigate the excited states for phenylsulfonylcarbazole compounds during the development of thermally activated delayed fluorescence (TADF) emitters for organic light emitting diode (OLED) applications.^20^ These studies suggest that the VQD method could be employed in the research and development of functional materials such as light emitting devices,^21^ photocatalysts,^22^ photovoltanic cells,^23^ and photochromic materials.^24^

In VQD calculations, the excited states are sequentially computed in conjunction with minimization of the defined cost function.^18^ Including penalty terms within the cost function is the most general means of ensuring that the computed wave function will be orthogonal to other states and have the desired eigenvalues.^13,18,25,26^ Penalty terms are introduced to raise the cost in the case that ansatz states are nonorthogonal to other states or eigenvalues deviate from user-specified values. Thus, the weighting coefficients that determine the magnitudes of the penalty terms dictate whether the desired excited states are obtained or not, and so these coefficients must be carefully selected. Even so, the coefficients are typically chosen heuristically. Although small coefficients are preferable so as to obtain rapid convergence, undesired excited states can possibly be obtained if the coefficients are too small. Conversely, if the coefficients are too large, the optimization may not converge properly. To address this problem, Kuroiwa and Nakagawa proposed a convenient formula to estimate the weighting coefficients as a part of their systematic analysis of the penalty terms.^27^ Assuming that this formula is generally applicable to calculations of the excited states for common molecules, it could greatly increase the practical applications of the VQD method. Although this formula was initially assessed on the basis of calculations of simple model systems such as H_2_ and H_4_, it should be examined to determine if it is more widely applicable to the excited states for more complex molecules. A practical procedure for utilizing this method should also be explored to prepare for upcoming applications of the VQD method using quantum computers.

On this basis, the present study applied the VQD method to calculate the core-excited and core-ionized states for common molecules based on simulated quantum circuit computations with a classical computer. An analysis using a classical computer is advantageous for this study because the results can be examined without being influenced by noise. The absorption bands derived from the core-excited state calculations were experimentally verified using X-ray absorption near edge spectroscopy (XANES),^28−31^ while the core-ionization energies were confirmed using X-ray photoelectron spectroscopy (XPS).^32−35^ It should be noted that the core-ionization energy is also referred to as the core–electron binding energy (CEBE) and that XPS is also traditionally known as electron spectroscopy for chemical analysis (ESCA). These spectroscopic techniques are widely utilized for the analysis of molecular structures and to determine the surface compositions of condensed materials, local bonding environments of specific atomic species, and electronic properties such as oxidation states. Computations of core-level states are of interest with regard to fundamental scientific studies and also with respect to the theoretical analyses of spectra.^36,37^ If the calculation of core-level states using the VQD method is found to be possible, this technique could have practical applications in conjunction with experimental analyses. As part of this study, the effects of the weighting coefficients used in the penalty terms were systematically analyzed by calculating the O 1s core-ionized state for a water molecule. The core-excitation energies and core-ionization energies for this molecule are on the order of hundreds of eV or higher and so are much larger than those of the more well-studied valence excited states. Therefore, the correlation of the weighting coefficients with the results could be clearly visualized. As examples of applications to functional materials, the O 1s core-excited states and Ti 2p core-ionized states for titanium dioxide (TiO_2_)^38−41^ and nitrogen-doped TiO_2_ (N-TiO_2_)^42,43^ were also calculated. These materials were selected because anatase TiO_2_ is well-known for its photocatalytic activity and N-TiO_2_ is a visible-light-sensitive photocatalyst.

Note that singly core-excited and singly core-ionized states were calculated in this study. Because these states have less multiconfiguration character, their wave functions can be reasonably described using a small number of electron configurations. Thus, even though only a small number of qubits can be currently employed in simulations on classical computers (as with calculations using actual NISQ devices), the excitation and ionization energies in such cases can be evaluated with high accuracy so that the calculation results can be verified by comparison with experimental data.^44^ It is noteworthy that Bauman at al. recently calculated the doubly excited core-level states for a water molecule using the QPE algorithm.^45^

## 2 Theory

### 2.1 VQD Method and Weighting Coefficients of Penalty Terms

Here, the VQD algorithm is briefly reviewed^18^ as well as the penalty terms related to the cost function.^27^ In the VQD algorithm, the *k*th ansatz state, |ψ(θ_*k*_)⟩, is calculated by minimizing the cost function
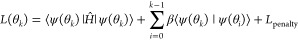
1Here *Ĥ* is a Hamiltonian written as *Ĥ* = ∑*c*_*j*_*P̂*_*j*_, where *P̂*_*j*_ is a single-qubit Pauli operator and *c*_*j*_ is the corresponding coefficient. We denote the eigenstates up to the (*k* – 1) th as |ψ(θ_*i*_)⟩ (*i* = 0, 1, ..., *k* – 1) and assume that |ψ(θ_*i*_)⟩ has been obtained before the calculation of |ψ(θ_*k*_)⟩. The ground state, |ψ(θ_0_)⟩, is initially obtained using the VQE method, after which the |ψ(θ_1_)⟩ state is calculated. In a similar manner, the |ψ(θ_2_)⟩, |ψ(θ_3_)⟩, ... and |ψ(θ_*k*_)⟩ states are calculated one by one in a sequential fashion. The second term of eq 1 is added to ensure that |ψ(θ_*k*_)⟩ is orthogonal to |ψ(θ_*i*_)⟩, while β is a weighting coefficient referred to as the overlap weight. Unless |ψ(θ_*k*_)⟩ is orthogonal to one of the |ψ(θ_*i*_)⟩ states, *L*(θ_*k*_) is increased by β because of this second term. The third term, *L*_penalty_, is introduced to impose a constraint on the spin multiplicity, spin quantum number and number of electrons associated with |ψ(θ_*k*_)⟩. The *L*_penalty_ adopted in this study consisted of three terms, written as
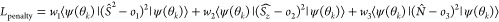
2where *Ŝ*^2^ is the total spin-squared operator, *Ŝ*_*z*_ is the *z*-component of the total spin operator, *Ŝ*, *N̂* is the particle-number operator, *w*_1_, *w*_2_, and *w*_3_ are weighting coefficients termed the s2 number weight, sz number weight, and particle number weight, respectively, and *o*_1_, *o*_2_, and *o*_3_ are the eigenvalues of *Ŝ*^2^, *Ŝ*_*z*_, and *N̂*, respectively, that must be satisfied by |ψ(θ_*k*_)⟩. Here, the *o*_*x*_ values (*x* = 1–3) are input parameters. *L*_penalty_ goes to zero when the eigenvalues of |ψ(θ_*k*_)⟩ coincide with the user-specified *o*_*x*_ values. In contrast, if the eigenvalues of |ψ(θ_*k*_)⟩ deviate from the *o*_*x*_ values, *L*(θ_*k*_) increases because *L*_penalty_ is non-zero. When the operators in eq 2 act on |ψ(θ_*k*_)⟩, we have
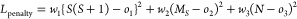
3where *S* is the total spin angular momentum, *M*_S_ is the spin quantum number, and *N* is the number of electrons. If we define Δ_*x*_ as the deviation of the eigenvalues of |ψ(θ_*k*_)⟩ from *o*_*x*_, we obtain
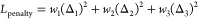
4where Δ_1_ = *S*(*S* + 1) – *o*_1_, Δ_2_ = *M*_*S*_ – *o*_2_, and Δ_3_ = *N* – *o*_3_.

### 2.2 Estimation of Weighting Coefficients for Excited-State Calculations

#### 2.2.1 Estimation of β

If |ψ(θ_*k*_)⟩ is orthogonal to |ψ(θ_*i*_)⟩ and all the Δ_*x*_ values are zero, *L*(θ_*k*_) is equal to the energy of |ψ(θ_*k*_)⟩, meaning that
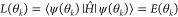
5

Next, we assume an ansatz state |ψ(θ_k_^*^)⟩ that is nonorthogonal to one of the |ψ(θ_*i*_)⟩ states. Consequently, the Δ_*x*_ values of |ψ(θ_k_^*^)⟩ are zero and its cost function, *L*(θ_k_^*^), can be written as
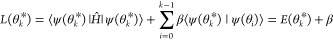
6if *L*(θ_*k*_) > *L*(θ_k_^*^), |ψ(θ_k_^*^)⟩ is obtained instead of |ψ(θ_*k*_)⟩. To obtain |ψ(θ_*k*_)⟩, β must satisfy the condition
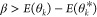
7so that *L*(θ_k_^*^) > *L*(θ_*k*_). The right-hand side of eq 7 is the energy gap between |ψ(θ_*k*_)⟩ and |ψ(θ_k_^*^)⟩, which is maximized if |ψ(θ_k_^*^)⟩ is the ground state: |ψ(θ_0_)⟩. We then have the condition for β written as
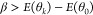
8

This condition was provided in the original paper in which VQD was described.^18^ Unfortunately, the right-hand side of eq 8 is the excitation energy of |ψ(θ_*k*_)⟩, which is a part of the calculation results. As a practical alternative, the excitation energy of the singly excited state can be estimated from the energy gap between the occupied and virtual orbitals related to the excitation as
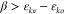
9where ε_*ko*_ and ε_*kv*_ are the energy levels of the occupied and virtual orbitals that are relevant to the main configuration of |ψ(θ_*k*_)⟩, respectively.

In this study, the β values were estimated based on eq 9, while the ε_*ko*_ and ε_*kv*_ values were obtained through the Hartree–Fock calculation that was used to obtain an initial state for the ground-state VQE calculation. In general, the gap between ε_*kv*_ and ε_*ko*_ will be larger than that between *E*(θ_*k*_) and *E*(θ_0_) when determined using the Hartree–Fock method.^46^ Consequently, the β value estimated from eq 9 should satisfy eq 8. Note that the value of ε_*kv*_ – ε_*ko*_ based on the use of Kohn–Sham orbitals may be smaller than *E*(θ_*k*_) – *E*(θ_0_). In addition, calculations using the density functional theory (DFT) method adopting an exchange-correlation functional tend to give a smaller energy gap between the occupied and virtual orbitals compared with the corresponding excitation energy.^46^

In this study, we adopted a procedure using the orbital energies of the ground state to estimate β and other weighting coefficients as mentioned above and below. As a matter of course, more sophisticated methods can also be applied to estimate the excitation energies. For example, Kuroiwa and Nakagawa used the configuration interaction singles and doubles (CISD) method to compute the excitation energies.^27^ The estimation based on the orbital energies of cationic species may also be practically useful.^47,48^

#### 2.2.2 Estimation of *w*_1_

The values of *w*_*x*_ (*x* = 1–3) were estimated using the formula proposed by Kuroiwa and Nakagawa.^27^ These calculations assumed |ψ(θ_k_^*^)⟩ with Δ_*p*_ ≠ 0 and Δ_*q*_ = Δ_*r*_ = 0, where (*p*, *q*, *r*) ∈ *x*, *p* ≠ *q*, *p* ≠ *r* and *q* ≠ *r*. In addition, |ψ(θ_k_^*^)⟩ was orthogonal to |ψ(θ_*i*_)⟩ because Δ_*p*_ ≠ 0. In this case, *L*(θ_k_^*^) could be written as
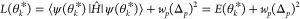
10

According to eqs 5 and 10, to have *L*(θ_k_^*^) > *L*(θ_*k*_)
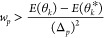
11must be true. Therefore, the lower limit of *w*_*p*_ is obtained when |Δ_*p*_| is at a minimum, |Δ_*p*_^min^|, such that
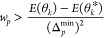
12where Δ_*p*_^min^ is the minimum deviation of the eigenvalue from the user-specified *o*_*p*_. Equation 12 indicates that the lower limit of *w*_*p*_ can be estimated based on *E*(θ_*k*_) – *E*(θ_k_^*^) and Δ_*p*_^min^.

We also assumed that, in the case of *p* = 1, |ψ(θ_k_^*^)⟩ has Δ_1_ ≠ 0 and Δ_2_ = Δ_3_ = 0 and |ψ(θ_k_^*^)⟩ is orthogonal to |ψ(θ_*i*_)⟩ when Δ_1_ ≠ 0. This gives a *L*(θ_k_^*^) value equal to
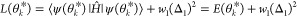
13

Because the eigenvalue of *Ŝ*^2^ is *S*(*S* + 1) and the minimum change in *S* is 1/2, |Δ_1_^min^| is 3/4. Hence, according to eq 12
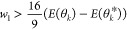
14should be true. The right-hand side of eq 14 is maximized when *E*(θ_k_^*^) = *E*(θ_0_), such that
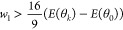
15

The energy gap between *E*(θ_*k*_) and *E*(θ_0_) is the excitation energy for *E*(θ_*k*_), which can be approximated based on ε_*kv*_ – ε_*ko*_ in a similar manner to eq 9 as
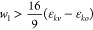
16

In the present work, eq 16 was adopted as the condition for *w*_1_.

#### 2.2.3 Estimation of *w*_2_

We assumed the case of *p* = 2, meaning |ψ(θ_k_^*^)⟩ with Δ_2_ ≠ 0 and Δ_1_ = Δ_3_ = 0 and |ψ(θ_k_^*^)⟩ orthogonal to |ψ(θ_*i*_)⟩ when Δ_2_ ≠ 0. *L*(θ_k_^*^) in this case is
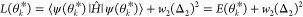
17

Since the eigenvalue of  is *M*_*S*_ and the minimum change in *M*_S_ is ±1/2, |Δ_2_^min^| is 1/2. According to eq 12, the *w*_2_ value should satisfy *w*_2_ > 4(*E*(θ_*k*_) – *E*(θ_k_^*^)) so that *L*(θ_k_^*^) > *L*(θ_*k*_). The gap between *E*(θ_*k*_) and *E*(θ_k_^*^) is maximized when *E*(θ_k_^*^) = *E*(θ_0_), and the gap between *E*(θ_*k*_) and *E*(θ_0_) can be approximated by ε_*kv*_ – ε_*ko*_ as with eq 9, such that
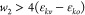
18

The *w*_2_ values in this study were estimated on the basis of eq 18.

#### 2.2.4 Estimation of *w*_3_

We assumed the case of *p* = 3, meaning |ψ(θ_k_^*^)⟩ with Δ_3_ ≠ 0 and Δ_1_ = Δ_2_ = 0 and |ψ(θ_k_^*^)⟩ orthogonal to |ψ(θ_*i*_)⟩ when Δ_3_ ≠ 0. *L*(θ_k_^*^) in this case is
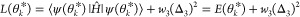
19

Because the eigenvalue of  is the number of electrons, its minimum change is ±1 and, accordingly, |Δ_3_^min^| is 1. Therefore, the *w*_3_ value should satisfy *w*_3_ > *E*(θ_*k*_) – *E*(θ_k_^*^) so that *L*(θ_k_^*^) > *L*(θ_*k*_). The gap between *E*(θ_*k*_) and *E*(θ_k_^*^) is maximized when *E*(θ_k_^*^) = *E*(θ_0_), and the gap between *E*(θ_*k*_) and *E*(θ_0_) can be approximated by ε_*kv*_ – ε_*ko*_. Thus, we have
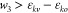
20

The *w*_3_ values in this study were estimated on the basis of eq 20. Overall, the weighting coefficients for the calculations of core-excited states were estimated so that
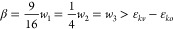
21

### 2.3 Estimation of Weighting Coefficients for Core-Ionized State Calculations

In the present study, each core-ionized state was calculated as a high-energy excited state for a molecule in its ionized state. In this procedure, the valence-ionized state was obtained as |ψ(θ_0_)⟩ via the VQE calculations. Accordingly, the charge-neutral ground state having a lower energy than |ψ(θ_0_)⟩ could be obtained as |ψ(θ_k_^*^)⟩ and *L*(θ_k_^*^) in this case was
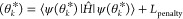
22

If |ψ(θ_*k*_)⟩ is the core-ionized state, *L*_penalty_ should satisfy the condition
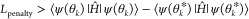
23such that *L*(θ_k_^*^) > *L*(θ_*k*_).

The right-hand side of eq 23 is the core-ionization energy resulting from the VQD calculation. This energy can be approximated as the negative of the core-orbital energy (ε_*ko*_) according to Koopman’s theorem^49^
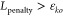
24

Equation 24 indicates that the weighting coefficients used in calculations of core-ionized states should be estimated based on – ε_*ko*_ instead of ε_*kv*_ – ε_*ko*_, in contrast to the calculations for the excited states. Accordingly, this work adopted the condition
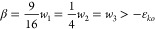
25and the ε_*ko*_ values obtained from the Hartree–Fock calculations were used.

## 3 Computational Details

Optimizations of molecular geometries via coupled cluster singles, doubles, and perturbative triples (CCSD(T))^50−52^ calculations and DFT calculations with the B3LYP functional^53,54^ were carried out using the Gaussian 09 program.^55^ The VQE and VQD calculations with the optimized structures were performed using the Qamuy program version 0.26.1^56^ in conjunction with a hardware-efficient ansatz.^57^ The Broyden–Fletcher–Goldfarb–Shanno (BFGS) algorithm was used to update variational parameters on a classical computer,^58^ while the Jordan–Wigner transformation was used to map Hamiltonians into a qubit circuit.^59,60^ The depth of the simulated quantum circuit (*D*) was given a value of 10. Noise was not considered with regard to the simulated quantum circuit, meaning that exact expected values were used in the simulations.

Because twice as many qubits as the number of active orbitals were required within the framework detailed above, the computational cost of the simulated calculations on a classical computer increased rapidly with increasing number of active orbitals. Accordingly, the active space of the VQD calculations had to be constructed using as few active orbitals as possible. In addition, the molecular orbitals were greatly perturbed by core hole formation. A core hole attracts electrons, leading to radial contraction of the outer orbitals, such that the core-hole state becomes highly stable. This effect is referred to as orbital relaxation,^61^ and the effects of orbital relaxation were not fully incorporated into the CI calculations using a limited number of electron configurations. In such cases, orbital optimization can be an effective means of improving the accuracy of the calculations,^44^ and so VQD calculations with and without molecular orbital optimization were compared. The calculations with and without orbital optimization corresponded to conventional complete active space self-consistent field (CASSCF)^62,63^ and complete active space configuration interaction (CASCI) calculations,^64^ respectively. A flexible basis set was also required to deal with the significant changes of the orbitals and Dunning’s cc-VXZ basis set (X = D, T, Q) was adopted.^65−67^ The weighting coefficients were estimated based on the Hartree–Fock orbital energies with the cc-pVDZ basis set.

### 3.1 Calculations of Core-Excited States for CO, H_2_CO, and HCN

The 1s → π* core-excited states for CO, H_2_CO, and HCN molecules were calculated. The molecular geometries were optimized using the CCSD(T)/cc-pVQZ approach and the core-excited states were calculated using the VQD method with the cc-pVXZ basis set. The 1s → π* core-excitation energies were obtained as the energy gaps between the ground and core-excited states. In the case of CO and H_2_CO, both the C 1s and O 1s core-excited states were calculated. Similarly, both the C 1s and N 1s core-excited states were calculated for HCN. The 1s and π* orbitals were selected as the active orbitals, and two electrons in these orbitals were treated as active. In the following discussion, the CAS constructed from these active orbitals and electrons is denoted as CAS(2e, 2o). During the calculations for CO and HCN, two 1s → π* core-excited states were found to be degenerate because two π* orbitals had the same energy level, and so only one state was calculated. For H_2_CO, the π* orbitals were split into different energy values because of the lower symmetry of the molecule. The weighting coefficients were estimated from the energy gaps between the 1s and π* orbitals in conjunction with eq 21.

### 3.2 Calculations of Core-Ionized States for CH_4_, NH_3_, H_2_O, and FH

The 1s core-ionized states for CH_4_, NH_3_, H_2_O, and FH molecules were calculated. The molecular geometries were optimized using the CCSD(T)/cc-pVQZ method, and the core-excited states were calculated using the VQD method with the cc-pVXZ basis set. The 1s core-ionization energies were obtained as the energy gaps between the charge-neutral (closed-shell) singlet ground state and doublet core-ionized state. The 1s orbital and the highest occupied orbital (HOMO) were selected as the active orbitals and three electrons in these orbitals were treated as active electrons. Henceforth, the CAS constructed from these active orbitals and electrons is denoted as CAS(3e, 2o). When adopting CAS(3e, 2o) as the configuration space, the valence-ionized state having the configuration (1s)^2^(HOMO)^1^ was the ground state and the core-ionized state was the excited state having the primary configuration (1s)^1^(HOMO)^2^. Accordingly, the core-ionized states could be calculated using the VQD algorithm. In a full CI calculation using a quantum computer with a larger number of qubits, all the occupied and virtual orbitals would be treated as active orbitals, such that the core-ionized states would be obtained as the excited states. The calculations presented herein based on CAS(3e, 2o) simulated this type of full CI calculation. The weighting coefficients for the VQD calculations were estimated based on the 1s orbital energies and using eq 25.

### 3.3 Effects of the Weighting Coefficients

The relationship between the weighting coefficients and the ansatz states was examined by calculating the 1s core-ionized state for a H_2_O molecule while varying the weighting coefficients. The molecular structure was optimized via a CCSD(T)/cc-pVQZ calculation while VQD calculations were carried out using the cc-pVDZ basis set.

### 3.4 Application to the TiO_2_ and N-TiO_2_ Models

#### 3.4.1 Preparation of the Models

In the quantum-chemical calculations of solid materials such as metals and metal oxides, a small cluster of atoms is often employed as a model.^68^ The calculation results obtained using this model cluster approach are significantly affected by the size and shape of the cluster, as well as the manner in which dangling bonds are terminated, all of which are arbitrarily assigned. Nakatsuji et al. proposed three principles for the construction of clusters: neutrality, stoichiometry, and coordination.^69^ In this work, we adopted a (TiO_2_)_16_ cluster, which is known to represent the smallest cluster of anatase-type TiO_2_ that satisfies these three conditions.^70^ Specifically, this cluster is charge neutral, has a stoichiometric Ti:O ratio of 1:2 (equal to that of the bulk system), and has no dangling bonds. The coordination numbers of the titanium atoms in this cluster were four or more, while those of the oxygen atoms were two or three. We initially prepared a (TiO_2_)_16_ cluster and optimized the geometrical structure using the B3LYP/cc-pVDZ basis set. Following this, a smaller cluster with one titanium atom and four oxygen atoms was cut out from the surface of this (TiO_2_)_16_ cluster and its dangling bonds were terminated with hydrogen atoms to obtain Ti(OH)_4_. The positions of the four hydrogen atoms in the Ti(OH)_4_ were reoptimized using the B3LYP/cc-pVDZ basis set, holding the other atoms at fixed positions during the reoptimization. Finally, one of the oxygen atoms of Ti(OH)_4_ was replaced with a nitrogen atom and the newly generated dangling bond on the nitrogen atom was saturated with a hydrogen atom to obtain Ti(OH)_3_(NH_2_). The positions of the hydrogen atoms of Ti(OH)_3_(NH_2_) were reoptimized using the B3LYP/cc-pVDZ basis set with the positions of the other atoms fixed. The resulting Ti(OH)_4_ and Ti(OH)_3_(NH_2_) clusters were employed as models of TiO_2_ and N-TiO_2_, respectively.

#### 3.4.2 Calculation of Valence Excited States, O 1s Core-Excited States, and Ti 2p Core-Ionized States

The HOMO → LUMO excited states, O 1s → LUMO core-excited states and Ti 2p core-ionized states for Ti(OH)_4_ and Ti(OH)_3_NH_2_ were calculated utilizing the VQD algorithm. The HOMO and LUMO of a solid material correspond to the valence band maximum (VBM) and the conduction band minimum (CBM), respectively. Therefore, absorption band edge shifts can be estimated from HOMO → LUMO excitation energies. The peak with the lowest energy in the O K-edge XANES spectrum was derived from the O 1s → LUMO excitation, and the corresponding peak in the XPS spectrum was situated at the Ti 2p core-ionization energy. The effects of nitrogen doping on these spectroscopic features could be examined based on calculations involving the Ti(OH)_4_ and Ti(OH)_3_(NH_2_) cluster models.

The CAS for the calculations of the HOMO → LUMO excited states was constructed using these two orbitals and the two electrons in the HOMO. Similarly, the CAS for the calculations of the O 1s → LUMO excited states was constructed using these two orbitals and the two electrons in the O 1s orbital. The CAS adopted for these calculations is denoted herein as CAS(2e, 2o). During the calculations of the Ti 2p core-ionized states, one of the Ti 2p orbitals and the HOMO were selected as the active orbitals and three electrons in these orbitals were treated as active electrons. This CAS is denoted as CAS(3e, 2o). Since there are three Ti 2p orbitals, three VQD calculations were made for each model, employing a different Ti 2p orbital as an active orbital.

The energy levels of the Ti 2p core-ionized states are split into 2p_1/2_ and 2p_3/2_ levels because of spin–orbit interactions.^71^ Unfortunately, the ab initio algorithm for relativistic effects is not presently implemented in the Qamuy program, and so this splitting had to be approximated. Although the gap between the 2p_1/2_ and 2p_3/2_ states (Δ_SO_) of Ti varies depending on the material, the value is typically within the narrow range of 5.60–6.13 eV,^72−74^ and so the Δ_SO_ value was assumed to be 5.7 eV in the present work.^73^ In the case of the core-ionized doublet state for an atom, the 2p_1/2_ energy level will be lower by (3/2)Δ_SO_ than the 2p level, while the 2p_3/2_ energy level will be higher by (1/2)Δ_SO_ (Figure S1). Consequently, the core-ionization energies of Ti(OH)_4_ and Ti(OH)_3_NH_2_, *E*_2p1/2_ and *E*_2p3/2_ derived from the 2p_1/2_ and 2p_3/2_ states, respectively, were estimated as
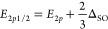
26and
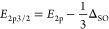
27where *E*_2p_ is the Ti 2p core-ionization energy calculated using the VQD method.

It should be noted that calculations using cluster models provide values for gas phase molecules, while the experimental values for TiO_2_ and N-TiO_2_ have been obtained using solid materials. When determining the ionization energies of finite systems such as molecules, the vacuum level is usually chosen as the reference. In contrast, the ionization energies of solids and surfaces are typically reported relative to the Fermi levels.^75−77^ Thus, the standard for the ionization energy will be different between molecules in the gas phase and solid materials. In addition, a core hole in a solid can be greatly stabilized by charge redistributions in the surrounding continuum, while such effects are absent in the gas phase.^78,79^ Therefore, the core-ionization energies that were calculated based on the cluster models could not be directly compared to the experimental data obtained from solids. For this reason, we also adjusted the calculated values so that the simulated *E*_2p_ value for Ti(OH)_4_ coincided with the experimental *E*_2p_ value for bulk TiO_2_ as estimated from the *E*_2p1/2_ and *E*_2p3/2_ values. It is noteworthy that theoretical methods for the calculation of absolute core ionization energies in solids from first-principles have been investigated in detail.^76−80^

## 4 Results and Discussion

### 4.1 Calculations of Core-Excited States for CO, H_2_CO, and HCN

The weighting coefficients used in the VQD calculations and the calculated core-excitation energies for CO, H_2_CO, and HCN molecules are presented in Table 1 with available experimental values.^81−84^ The 1s (ε_1s_) and π* (ε_π*_) orbital energies which were used for the estimation for weighting coefficients are summarized in Table S1. Because the ε_1s_ energies increased in the order of C 1s > N 1s > O 1s, the weighting coefficients were in the reverse order of O 1s > N 1s > C 1s. The 1s → π* core-excited states could be successfully calculated using the VQD method with these weighting coefficients. The calculated core-excitation energies deviated from the experimental values by only 3–5% in the case that Hartree–Fock orbitals were employed. Remarkably, these deviations were further reduced to less than 1.1% by incorporating orbital relaxations. In particular, discrepancies of less than 0.4% were achieved using the cc-pVTZ or cc-pVQZ basis set, meaning that the core-excitation energies calculated using the VQD method were in quantitatively good agreement with the experimental data. The CAS contained only the minimum configurations describing the wave function of the core-excited state, and so the results were highly dependent on whether or not orbital optimizations were applied. The calculated core-excitation energies of H_2_CO were lower than those of CO as well as the experimental values. The calculated core-excitation energies were also correlated with the local chemical-bonding states, indicating that the VQD calculations could be applied to structural analyses based on comparisons with experimental XANES spectra.

**Table 1:** Weighting Coefficients in the Cost Function for the VQD Calculations: Overlap Weights (β), s2 Number Weights (*w*_1_), sz Number Weights (*w*_2_) and Particle Number Weights (*w*_3_), and Calculated 1s → π* Core Excitation Energies for CO, H_2_CO, and HCN^a^

		weighting coefficients (hartrees)				core excitation energy (eV)					
molecule	core orbital	β	*w*_1_	*w*_2_	*w*_3_		basis set	without orbital optimization		with orbital optimization	
CO	C 1s	12.0	21.4	48.0	12.0	calc	cc-pVDZ	298.39	(+3.82%)	290.47	(+1.07%)
							cc-pVTZ	298.79	(+3.96%)	288.39	(+0.34%)
							cc-pVQZ	299.50	(+4.21%)	288.10	(+0.24%)
						exptl^b^				287.4	
	O 1s	21.0	37.4	84.0	21.0	calc	cc-pVDZ	552.92	(+3.52%)	536.14	(+0.38%)
							cc-pVTZ	553.25	(+3.59%)	533.97	(−0.03%)
							cc-pVQZ	553.74	(+3.68%)	533.57	(−0.10%)
						exptl^c^				534.1	
H_2_CO	C 1s	12.0	21.4	48.0	12.0	calc	cc-pVDZ	297.99	(+4.34%)	288.54	(+1.03%)
							cc-pVTZ	298.20	(+4.41%)	286.58	(+0.34%)
							cc-pVQZ	298.73	(+4.60%)	286.37	(+0.27%)
						exptl^d^				285.6	
	O 1s	21.0	37.4	84.0	21.0	calc	cc-pVDZ	548.83	(+3.40%)	532.95	(+0.41%)
							cc-pVTZ	549.35	(+3.50%)	530.82	(+0.00%)
							cc-pVQZ	549.88	(+3.60%)	530.45	(−0.07%)
						exptl^d^				530.8	
HCN	C 1s	12.0	21.4	48.0	12.0	calc	cc-pVDZ	299.36	(+4.53%)	289.38	(+1.04%)
							cc-pVTZ	299.55	(+4.59%)	287.27	(+0.30%)
							cc-pVQZ	300.15	(+4.80%)	287.01	(+0.21%)
						exptl^e^				286.4	
	N 1s	16.0	28.5	64.0	16.0	calc	cc-pVDZ	415.33	(+3.91%)	402.53	(+0.71%)
							cc-pVTZ	415.78	(+4.02%)	400.28	(+0.15%)
							cc-pVQZ	416.49	(+4.20%)	399.97	(+0.07%)
						exptl^e^				399.7	

a Available experimental values are also shown. The values in parentheses are deviations from the experimental values.

b Reference (81).

c Reference (82).

d Reference (83).

e Reference (84).

The calculated core-excitation energies were reduced depending on the basis set in the order of cc-pVDZ > cc-pVTZ > cc-pVQZ when orbital optimizations were carried out. A larger basis set was advantageous because it allowed orbital relaxation effects on core hole formation to be incorporated, thus reducing the energy of the core-excited state relative to that of the ground state. This obvious effect of the basis set size on the core-excitation energy was not observed in calculations performed without orbital optimization. Thus, the effects of enlarging the basis set were also fully incorporated in association with orbital optimization. Nevertheless, the calculated core-excitation energies deviated from the experimental values even when the larger cc-pVTZ and cc-pVQZ basis sets were used. The remaining discrepancies can be attributed to the lack of dynamical correlation within the present calculations.

### 4.2 Calculations of Core-Ionized States for CH_4_, NH_3_, H_2_O, and FH

The weighting coefficients used in the VQD calculations and the calculated core-ionization energies are collected in Table 2 along with the available experimental data.^85^ The ε_1s_ energies for CH_4_, NH_3_, H_2_O, and FH molecules used for the estimation of the weighting coefficients are presented in Table S2. The weighting coefficients decreased in the order of FH > H_2_O > NH_3_ > CH_4_ because the ε_1s_ energies were in the order of C 1s > N 1s > O 1s > F 1s. By adopting these weighting coefficients, the 1s core-ionized states were successfully calculated as |ψ(θ_1_)⟩. The resulting energies were found to deviate from the experimental values by 3.01–4.24% when orbital optimization was not applied, although these discrepancies were significantly reduced (to less than 1%) by carrying out orbital optimizations. The core-ionization energies obtained using the VQD method were in quantitatively good agreement with the experimental data in the case that orbital optimizations were employed, as was also the case for the core-excited state calculations. These results indicate that the peak position in an XPS spectrum can be predicted with reasonable accuracy using VQD calculations.

**Table 2:** Weighting Coefficients in the Cost Function for the VQD Calculations: Overlap Weights (β), s2 Number Weights (*w*_1_), sz Number Weights (*w*_2_), and Particle Number Weights (*w*_3_), and Calculated Core Ionization Energies for CH_4_, NH_3_, H_2_O, and FH^a^

	weighting coefficients (hartrees)				core ionization energy					
molecule	β	*w*_1_	*w*_2_	*w*_3_		basis set	without orbital optimization		with orbital optimization	
CH_4_	12.0	21.4	48.0	12.0	calc	cc-pVDZ	302.23	(+3.91%)	290.51	(−0.12%)
						cc-pVTZ	302.05	(+3.85%)	288.35	(−0.86%)
						cc-pVQZ	302.15	(+3.88%)	288.20	(−0.92%)
					exptl^b^				290.86	
NH_3_	16.0	28.5	64.0	16.0	calc	cc-pVDZ	422.77	(+4.24%)	407.63	(+0.51%)
						cc-pVTZ	422.70	(+4.22%)	405.64	(+0.02%)
						cc-pVQZ	422.78	(+4.24%)	405.41	(−0.04%)
					exptl^b^				405.57	
H_2_O	21.0	37.4	84.0	21.0	calc	cc-pVDZ	559.21	(+3.58%)	541.64	(+0.33%)
						cc-pVTZ	559.32	(+3.61%)	539.60	(−0.05%)
						cc-pVQZ	559.46	(+3.63%)	539.27	(−0.11%)
					exptl^b^				539.86	
FH	27.0	48.0	108.0	27.0	calc	cc-pVDZ	715.05	(+3.01%)	695.84	(+0.24%)
						cc-pVTZ	715.28	(+3.04%)	693.63	(−0.08%)
						cc-pVQZ	715.41	(+3.06%)	693.20	(−0.14%)
					exptl^b^				694.18	

a Available experimental values are also shown. The values in parentheses are deviations from the experimental values.

b Reference (86).

The calculated core-ionization energies decreased with increasing size of the adopted basis set when applying orbital optimization, in the order of cc-pVDZ > cc-pVTZ > cc-pVQZ. This ordering suggests that orbital relaxation, along with core-hole formation, could be more accurately described using a more flexible basis set, leading to a downward shift of the energy level of the core-ionized state relative to that of the neutral ground state.^44^ The core-ionization energies calculated without orbital optimization were not clearly correlated with the size of the basis set because the effects of the orbital relaxations were only insufficiently considered. The core-ionization energies were found to be generally underestimated in the case that larger basis sets such as the cc-pVTZ or cc-pVQZ sets were employed. It has also been reported that the core-ionization energy is typically underestimated in the framework of the Δ*S*CF method.^86^ Therefore, the deviations from the experimental values can be attributed to a lack of incorporated dynamical correlation. The charge-neutral ground state has one more electron than the core-ionized state, and so it is reasonable to expect that the extent of dynamical electron correlation in the charge-neutral ground state will be larger than that in the core-ionized state. Therefore, the gap between these states is underestimated when the dynamical correlation is not fully considered.

### 4.3 Effect of the Weighting Coefficients

**Figure 1:**
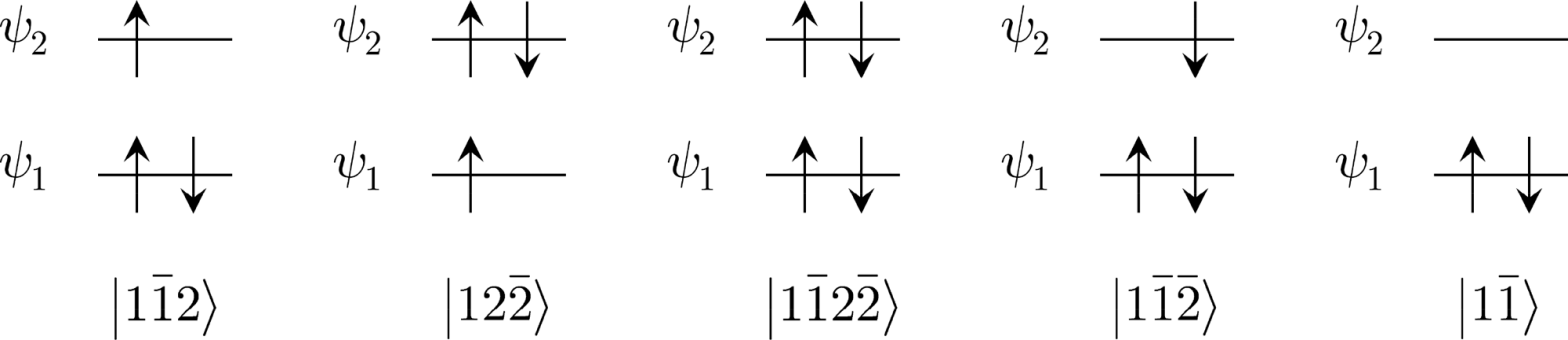
Primary configurations of the electronic states determined from VQD calculations of the core-ionized states for H_2_O. Here, ψ_1_ and ψ_2_ correspond to the O 1s orbital and HOMO, respectively.

The present calculations were carried out while varying the weighting coefficients, and Figure 1 shows the primary configurations of the electronic states obtained for the H_2_O molecule. The calculated electronic state energies, eigenvalues, and imposed penalties are provided in Table 3. Table 4 summarizes the calculated energies and the assignments of the first, second, and third electronic states: |ψ(θ_0_)⟩, |ψ(θ_1_)⟩, and |ψ(θ_2_)⟩ The costs of these states are summarized in Table 5. Below, we review the results with respect to each coefficient.

**Table 3:** Calculated Energies, Eigenvalues of the Observables, and Imposed Penalty Terms of |ψ_*k*_⟩ states (*k* ≥ 1) Obtained from VQD Calculations of the Core-Ionized States for H_2_O^a^

		eigenvalue			
electronic state	energy	*S*(*S* + 1)	*M*_*S*_	*N*	imposed penalty terms
|122̅⟩	–55.4760	3/4	1/2	3	0
|11̅2⟩	–75.5335	3/4	1/2	3	β
|11̅22̅⟩	–76.0268	0	0	4	(9/16)*w*_1_ + (1/4)*w*_2_ + *w*_3_
|11̅2̅⟩	–75.5335	3/4	–1/2	3	*w*_2_
|11̅⟩	–74.2798	0	0	2	(9/16)*w*_1_ + (1/4)*w*_2_ + *w*_3_

a Each electronic state is denoted by a Slater determinant that corresponds to its main configuration as shown in Figure 1.

**Table 4:** Electronic State Energies (*E*(θ_*k*_)) and Assignments of |ψ(θ_*k*_)⟩ for H_2_O as Calculated Using the VQD Method (*k* = 0, 1, and 2)^a^

					|ψ(θ_0_)⟩		|ψ(θ_1_)⟩		|ψ(θ_2_)⟩	
condition	β	*w*_1_	*w*_2_	*w*_3_	*E*(θ_0_)	assignment	*E*(θ_1_)	assignment	*E*(θ_2_)	assignment
(i)	15.0	37.4	84.0	21.0	–75.5335	|11̅2⟩	–75.5335	|11̅2⟩	–55.4760	|122̅⟩
	18.0	37.4	84.0	21.0	–75.5335	|11̅2⟩	–75.5335	|11̅2⟩	–55.4760	|122̅⟩
	21.0	37.4	84.0	21.0	–75.5335	|11̅2⟩	–55.4760	|122̅⟩	–75.5335	|11̅2⟩
	24.0	37.4	84.0	21.0	–75.5335	|11̅2⟩	–55.4760	|122̅⟩	–75.5335	|11̅2⟩
	27.0	37.4	84.0	21.0	–75.5335	|11̅2⟩	–55.4760	|122̅⟩	–75.5335	|11̅2⟩
(ii)	21.0	24.0	0.0	0.0	–75.5335	|11̅2⟩	–75.5335	|11̅2̅⟩	–76.0268	|11̅22̅⟩
	21.0	32.0	0.0	0.0	–75.5335	|11̅2⟩	–75.5335	|11̅2̅⟩	–76.0268	|11̅22̅⟩
	21.0	37.4	0.0	0.0	–75.5335	|11̅2⟩	–75.5335	|11̅2̅⟩	–55.4760	|122̅⟩
	21.0	40.0	0.0	0.0	–75.5335	|11̅2⟩	–75.5335	|11̅2̅⟩	–55.4760	|122̅⟩
	21.0	48.0	0.0	0.0	–75.5335	|11̅2⟩	–75.5335	|11̅2̅⟩	–55.4760	|122̅⟩
(iii)	21.0	0.0	60.0	0.0	–75.5335	|11̅2⟩	–76.0268	|11̅22̅⟩	–74.2798	|11̅⟩
	21.0	0.0	72.0	0.0	–75.5335	|11̅2⟩	–76.0268	|11̅22̅⟩	–74.2798	|11̅⟩
	21.0	0.0	84.0	0.0	–75.5335	|11̅2⟩	–55.4760	|122̅⟩	–76.0268	|11̅22̅⟩
	21.0	0.0	96.0	0.0	–75.5335	|11̅2⟩	–55.4760	|122̅⟩	–75.5335	|11̅2⟩
	21.0	0.0	108.0	0.0	–75.5335	|11̅2⟩	–55.4760	|122̅⟩	–75.5335	|11̅2⟩
(iv)	21.0	0.0	0.0	15.0	–75.5335	|11̅2⟩	–75.5335	|11̅2̅⟩	–76.0268	|11̅22̅⟩
	21.0	0.0	0.0	18.0	–75.5335	|11̅2⟩	–75.5335	|11̅2̅⟩	–76.0268	|11̅22̅⟩
	21.0	0.0	0.0	21.0	–75.5335	|11̅2⟩	–75.5335	|11̅2̅⟩	–55.4760	|122̅⟩
	21.0	0.0	0.0	24.0	–75.5335	|11̅2⟩	–75.5335	|11̅2̅⟩	–55.4760	|122̅⟩
	21.0	0.0	0.0	27.0	–75.5335	|11̅2⟩	–75.5335	|11̅2̅⟩	–55.4760	|122̅⟩
(v)	21.0	0.0	21.0	15.75	–75.5335	|11̅2⟩	–55.4760	|122̅⟩	–76.0268	|11̅22̅⟩
	21.0	7.0	21.0	11.8125	–75.5335	|11̅2⟩	–55.4760	|122̅⟩	–76.0268	|11̅22̅⟩
	21.0	14.0	21.0	7.875	–75.5335	|11̅2⟩	–55.4760	|122̅⟩	–76.0268	|11̅22̅⟩
	21.0	21.0	21.0	3.9375	–75.5335	|11̅2⟩	–55.4760	|122̅⟩	–76.0268	|11̅22̅⟩
	21.0	28.0	21.0	0.0	–75.5335	|11̅2⟩	–55.4760	|122̅⟩	–76.0268	|11̅22̅⟩

a All values are in hartrees.

**Table 5:** Calculated Electronic State Energies for |ψ(θ_*k*_)⟩ (*E*(θ_*k*_)), Cost Functions (*L*(θ_*k*_)), and *L*(θ_*k*_) – *E*(θ_*k*_) Values (*k* = 1, 2)^a^

					|ψ(θ_1_)⟩			|ψ(θ_2_)⟩		
condition	β	*w*_1_	*w*_2_	*w*_3_	*E*(θ_1_)	*L*(θ_1_)	*L*(θ_1_) – *E*(θ_1_)	*E*(θ_2_)	*L*(θ_2_)	*L*(θ_2_) – *E*(θ_2_)
(i)	15.0	37.4	84.0	21.0	–75.5335	–60.5335	15.0	–55.4760	–55.4760	0.0
	18.0	37.4	84.0	21.0	–75.5335	–57.5335	18.0	–55.4760	–55.4760	0.0
	21.0	37.4	84.0	21.0	–55.4760	–55.4760	0.0	–75.5335	–54.5335	21.0
	24.0	37.4	84.0	21.0	–55.4760	–55.4760	0.0	–75.5335	–51.5335	24.0
	27.0	37.4	84.0	21.0	–55.4760	–55.4760	0.0	–75.5335	–48.5335	27.0
(ii)	21.0	24.0	0.0	0.0	–75.5335	–75.5335	0.0	–76.0268	–62.5268	13.5
	21.0	32.0	0.0	0.0	–75.5335	–75.5335	0.0	–76.0268	–58.0268	18.0
	21.0	37.4	0.0	0.0	–75.5335	–75.5335	0.0	–55.4760	–55.4760	0.0
	21.0	40.0	0.0	0.0	–75.5335	–75.5335	0.0	–55.4760	–55.4760	0.0
	21.0	48.0	0.0	0.0	–75.5335	–75.5335	0.0	–55.4760	–55.4760	0.0
(iii)	21.0	0.0	60.0	0.0	–76.0268	–61.0268	15.0	–74.2798	–59.2798	15.0
	21.0	0.0	72.0	0.0	–76.0268	–58.0268	18.0	–74.2798	–56.2798	18.0
	21.0	0.0	84.0	0.0	–55.4760	–55.4760	0.0	–76.0268	–55.0268	21.0
	21.0	0.0	96.0	0.0	–55.4760	–55.4760	0.0	–75.5335	–54.5335	21.0
	21.0	0.0	108.0	0.0	–55.4760	–55.4760	0.0	–75.5335	–54.5335	21.0
(iv)	21.0	0.0	0.0	15.0	–75.5335	–75.5335	0.0	–76.0268	–61.0268	15.0
	21.0	0.0	0.0	18.0	–75.5335	–75.5335	0.0	–76.0268	–58.0268	18.0
	21.0	0.0	0.0	21.0	–75.5335	–75.5335	0.0	–55.4760	–55.4760	0.0
	21.0	0.0	0.0	24.0	–75.5335	–75.5335	0.0	–55.4760	–55.4760	0.0
	21.0	0.0	0.0	27.0	–75.5335	–75.5335	0.0	–55.4760	–55.4760	0.0
(v)	21.0	0.0	21.0	15.75	–55.4760	–55.4760	0.0	–76.0268	–55.0268	21.0
	21.0	7.0	21.0	11.8125	–55.4760	–55.4760	0.0	–76.0268	–55.0268	21.0
	21.0	14.0	21.0	7.875	–55.4760	–55.4760	0.0	–76.0268	–55.0268	21.0
	21.0	21.0	21.0	3.9375	–55.4760	–55.4760	0.0	–76.0268	–55.0268	21.0
	21.0	28.0	21.0	0.0	–55.4760	–55.4760	0.0	–76.0268	–55.0268	21.0

a All values are in hartrees.

With regard to condition (i), the core-ionized state represented by |122̅⟩ was obtained as the ansatz state |ψ(θ_1_)⟩ with β ≥ 21.0. In contrast, the state was |ψ(θ_2_)⟩ and the ground state |11̅2⟩ was obtained as both |ψ(θ_0_)⟩ and |ψ(θ_1_)⟩ with β < 21.0. In the case of β < 21.0, |ψ(θ_1_)⟩ was nonorthogonal to |ψ(θ_0_)⟩ and the penalty of β was imposed on its cost function. However, ⟨11̅2|*Ĥ*|11̅2⟩ + β was still less than the cost function for the core-ionized state |122̅⟩. Therefore, the ground state |11̅2⟩ was obtained again as |ψ(θ_1_)⟩. If |ψ(θ_2_)⟩ is also |11̅2⟩, its cost function will be ⟨11̅2|*Ĥ*|11̅2⟩ + 2β because |ψ(θ_2_)⟩ is nonorthogonal to both |ψ(θ_0_)⟩ and |ψ(θ_1_)⟩, and so the cost function will be greater than that of the core-ionized state |122̅⟩. Thus, the core-ionized state |122̅⟩ was obtained as |ψ(θ_2_)⟩. In the case with β ≥ 21.0, the cost function for |11̅2⟩ became greater than that for |122̅⟩. Consequently, |122̅⟩ was obtained as |ψ(θ_1_)⟩ and |11̅2⟩ was obtained as |ψ(θ_2_)⟩ in ascending order of the cost function.

For condition (ii), the ansatz state |ψ(θ_1_)⟩ was calculated to be |11̅2̅⟩ in all cases. Because *w*_2_ = 0 in this case, no penalty was imposed on the deviation of the spin quantum number. In addition, the |11̅2̅⟩ state is orthogonal to |11̅2⟩, resulting in no penalty being imposed by β. As a result, the cost function for |11̅2̅⟩ was the same as that for the ground state |11̅2⟩. Therefore, |11̅2̅⟩ was consistently obtained as |ψ(θ_1_)⟩ and other states were obtained as |ψ(θ_2_)⟩. In the case of *w*_1_ < 37.4, the charge neutral ground state |11̅22̅⟩ with four electrons was obtained as |ψ(θ_2_)⟩. Since the charge neutral ground state |11̅22̅⟩ is orthogonal to the ground state of the ionized state |11̅2⟩ and *w*_2_ = *w*_3_ = 0, only an *L*_*penalty*_ of (9/16)*w*_1_ was imposed on its cost function. Therefore, ⟨11̅22̅|*Ĥ*|11̅22̅⟩ + (9/16)*w*_1_ was less than the energy of the core ionized state |122̅⟩ for *w*_1_ < 37.4. The ordering of the |11̅22̅⟩ and |122̅⟩ cost functions was inverted in the case of *w*_1_ ≥ 37.4 and |122̅⟩ was obtained as |ψ(θ_2_)⟩.

The calculation results for condition (iii) were complicated. The calculations with *w*_2_ < 84.0 provided the charge-neutral ground state |11̅22̅⟩ as |ψ(θ_1_)⟩ and the valence-double-ionized state |11̅⟩ as |ψ(θ_2_)⟩. Both states are orthogonal to the ground state |11̅2⟩ and *w*_1_ = *w*_3_ = 0, such that an *L*_penalty_ of (1/4)*w*_2_ was imposed on |11̅22̅⟩. However, for *w*_2_ < 84.0, the *L*_penalty_ value was less than the energy gap between |11̅2⟩ and |122̅⟩ and so |11̅22̅⟩ was obtained as |ψ(θ_1_)⟩. The same penalty was imposed on |11̅⟩ for *w*_1_ = *w*_3_ = 0, and was less than the energy gap between |11̅⟩ and |122̅⟩. Consequently, |11̅⟩ was obtained as |ψ(θ_2_)⟩. In the case with *w*_2_ = 84.0, the core-ionized state |122̅⟩ was obtained as |ψ(θ_1_)⟩ and |11̅22̅⟩ was obtained as |ψ(θ_2_)⟩ because *L*(θ_1_) = ⟨122̅|*Ĥ*|122̅⟩ was less than *L*(θ_2_) = ⟨11̅22̅|*Ĥ*|11̅22̅⟩ + (1/4)*w*_2_. In the case of *w*_2_ > 84.0, the ground state |11̅2⟩ was obtained as |ψ(θ_2_)⟩ because ⟨11̅22̅|*Ĥ*|11̅22̅⟩ + (1/4)*w*_2_ became greater than ⟨11̅2|*Ĥ*|11̅2⟩ + β.

The results obtained using condition (iv) were similar to those observed for condition (ii). The |ψ(θ_1_)⟩ state was calculated to be |11̅2̅⟩ in all cases and, although |Δ_2_| = 1, no penalty was imposed because *w*_2_ = 0. In the case of *w*_3_ < 21.0, the neutral ground state |11̅22̅⟩ was obtained as |ψ(θ_2_)⟩ because its *L*_penalty_ of *w*_3_ was less than the energy gap between |11̅22̅⟩ and |122̅⟩. The core-ionized state |122̅⟩ was obtained as |ψ(θ_2_)⟩ for *w*_3_ ≥ 21.0, suggesting that the ordering of the cost function was inverted due to the increase in *w*_3_.

In conclusion, the weighting coefficients estimated from eqs 21 and 25 were adequate to raise the cost of undesired ansatz states so that the target state was obtained. In addition, the penalty terms imposed on the ansatz states shown in Table 3 suggest that fewer weighting coefficients were effective for this system. A penalty of *w*_2_ was imposed on |11̅2̅⟩, and so *w*_2_ = −ε_1s_ was sufficient to raise the cost function of |11̅2̅⟩ above that of |122̅⟩. Accordingly, (9/16)*w*_1_ + *w*_3_ > – (3/4)ε_1s_ was suitable for increasing the cost functions for |11̅22̅⟩ and |11̅⟩ such that they were higher than that for |122̅⟩. The calculations using the weighting coefficients meeting these requirements were carried out as condition (v). The core-ionized state |122̅⟩ and the neutral ground state |11̅22̅⟩ were obtained as |ψ(θ_1_)⟩ and |ψ(θ_2_)⟩, respectively. Thus, the lower limit of each weighting coefficient was dependent on the system. The weighting coefficients estimated from eqs 21 and 25 were based on the minimum changes in the eigenvalues;^27^ therefore, the estimated values were expected to be consistently equal to or larger than the lower limits that were required. The estimations based on eqs 21 or 25 are potentially effective for any construction of the active space. However, the results in Table 4 indicate that various electronic states other than the target state can be found in the calculations; the number of electronic states that can be found in the calculations rapidly increases with the active orbitals and electrons. Those states can be local minima and disturb the minimization of the cost function and its convergence. The influence of an expansion of the active space on the calculation results and the minimization process of the cost function should be clarified through further applications.

### 4.4 Calculation Results for the TiO_2_ and N-TiO_2_ Models

The optimized Cartesian coordinates for the models are summarized in Tables S3 and S4. The calculation results for the optimized structures are summarized in Table 6 and discussed below.

**Table 6:** Calculated HOMO → LUMO Excitation Energies, O 1s → LUMO Core-Excitation Energies, Calculated Ti 2p Core-Ionization Energies (*E*_2p_), and Estimated *E*_2p1/2_ and *E*_2p3/2_ Values for the Ti(OH)_4_ and Ti(OH)_3_(NH_2_) Models; Experimental Values Obtained for Bulk TiO_2_ and N-TiO_2_ Are Also Presented

		HOMO → LUMO excitation energy			Ti 2p core-ionized (eV)		
model	orbital	(eV)	(nm)	O 1s core-excitation energy (eV)	*E*_2p_	*E*_2p1/2_	*E*_2p3/2_
Ti(OH)_4_	not optimized	9.25	134.1	555.92	492.01	495.81	490.11
	optimized	3.21	386.3	532.41	477.42	481.22	475.52
Ti(OH)_4_ (shifted)					460.50^a^	464.30	458.60
TiO_2_ (exptl)		3.2^b^	387.4^b^	531.1^c^	460.5^d^	464.3,^e^ 464.5^f^	458.6,^e^ 458.7^f^
Ti(OH)_3_NH_2_	not optimized	7.67	161.7	556.36	491.06	494.86	489.16
	optimized	2.92	424.5	533.01	475.98	479.78	474.08
Ti(OH)_3_NH_2_ (shifted)					459.06^g^	462.86	457.16
N-TiO_2_ (exptl)		2.5^b^	495.9^b^	531.0–531.4^h^		464.0,^i^ 464.1^j^	458.0,^e^ 458.3^j^

a Shifted downward by 16.92 eV from the calculated values after orbital optimizations so that the calculated *E*_2p_ for Ti(OH)_4_ coincides with the experimental value of 460.5 eV.

b Data obtained from ref (42) based on the absorption band edge.

c Data taken from ref (87).

d Estimated from the experimental *E*_2p1/2_ and *E*_2p3/2_ values in ref (73) utilizing eqs 26 and (27.

e Data taken from ref (73).

f Data taken from ref (74).

g Shifted downward by 16.92 eV from the calculated values for Ti(OH)_3_(NH_2_) after orbital optimizations.

h Data taken from ref (87). The values were shifted upward with increasing amounts of nitrogen doping.

i Extracted from Figure 8b of ref (73).

j Extracted from Figure 2a of ref (74).

#### 4.4.1 HOMO → LUMO Excited States

The HOMO and LUMO energy levels and weighting coefficients used in the calculations of HOMO → LUMO excited states are summarized in Table S5. The ε_HOMO–LUMO_ values were rounded up to the first decimal place prior to being used for the estimation of weighting coefficients. Since the HOMO → LUMO excitation energies were much smaller than the core-excitation energies and core-ionization energies, the weighting coefficients for the calculations of HOMO → LUMO excited states were also smaller.

**Figure 2:**
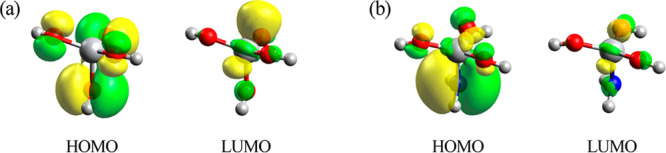
Images showing the HOMO and LUMO for (a) Ti(OH)_4_ and (b) Ti(OH)_3_(NH_2_) as calculated using the Hartree–Fock method.

The calculated excitation energies are presented in Table 6 along with the wavelengths converted from the excitation energies; the total electronic state energies are shown in Table S6. The HOMO → LUMO excited states corresponding to the S_1_ states were obtained as the |ψ(θ_1_)⟩ ansatz states using the weighting coefficients in Table S5. The calculated absorption wavelength for Ti(OH)_3_(NH_2_) was longer than that for Ti(OH)_4_, indicating that the absorption wavelength for TiO_2_ should be red-shifted following nitrogen doping, in qualitatively good agreement with experimental findings.^42,43^Figure 2 presents images of the HOMOs and LUMOs for the models as calculated using the Hartree–Fock method. The HOMO and LUMO for Ti(OH)_4_ were mainly derived from O 2p and Ti 3d orbitals, respectively. In contrast, the HOMO for Ti(OH)_3_(NH_2_) originated from the N 2p orbital. Because the energy level of the N 2p orbital is higher than that of the O 2p orbital (Table S5), the HOMO–LUMO gap is decreased by the substitution of an NH_2_ group for an OH group, which is analogous to the redshift of the absorption wavelength for bulk TiO_2_ as a result of nitrogen doping. The calculated HOMO → LUMO excitation energies were also greatly decreased following orbital optimization. The calculated energies for Ti(OH)_4_ and Ti(OH)_3_(NH_2_) were 3.21 and 2.92 eV, respectively, in good agreement with the experimentally observed band gaps of 3.2 eV for TiO_2_ and 2.5 eV for N-TiO_2_.^42^ These results suggest that the adopted models had similar electronic properties to those of the bulk systems, even though they were much smaller in size. It should also be noted that the calculation results obtained using a cluster model depend on the properties of the cluster and that dynamical correlations were not considered in the present VQD calculations. The errors derived from these conditions might counteract one another to provide successful results.

#### 4.4.2 O 1s Core-Excited States

The O 1s and LUMO energy levels and weighting coefficients used in the calculations of the O 1s → LUMO excited states are summarized in Table S7. Note that the ε_1s–LUMO_ value was rounded up to the first decimal place prior to being used for the estimation of the weighting coefficients. The calculated O 1s → LUMO excited state energies for the Ti(OH)_4_ and Ti(OH)_3_(NH_2_) models are provided in Table S8. These energies were averaged for the calculation of the core excitation energy value of each model, and the core excitation energy of Ti(OH)_3_(NH_2_) was found to be higher than that of Ti(OH)_4_ by approximately 0.6 eV (Table 6). These results suggest that the peak in the XANES spectrum of TiO_2_ originating from the O 1s → LUMO excitation was shifted toward higher energy as a result of nitrogen doping, in agreement with experimental observations.^87^ The core-excitation energies calculated with orbital optimization were generally in good agreement with the corresponding experimental values. The LUMOs shown in Figure 2 suggest that these orbitals primarily originated from Ti 3d orbitals and that their energy levels were shifted upward due to the substitution of a N atom for an O atom of Ti(OH)_4_ (Table S8). In this oxide, the Ti–O bond is polarized to Ti^δ+^–O^δ−^ because of the high electronegativity of the oxygen atom.^88^ Consequently, the electron density in the Ti atom is increased by the substitution of a N atom for an O atom because of the lower electronegativity of N compared with O. This effect, in turn, is responsible for the upward shift of the Ti 3d level.^74^ As a result, the core excitation energy of Ti(OH)_3_(NH_2_) can be higher than that of Ti(OH)_4_.

The calculated core-excitation energies were decreased by approximately 13 eV following orbital optimization, suggesting that significant orbital relaxation was associated with core-hole formation compared with HOMO–LUMO excitation. Although a hole in the HOMO primarily affects the electrons excited to the virtual orbitals, a core hole additionally affects the valence occupied orbitals, leading to significant relaxation. As a result, the calculated O 1s → LUMO core-excitation energies were greatly reduced through orbital optimization.

The calculated shift of the core-excitation energy due to the substitution of a N atom for an O atom was larger than the experimentally observed value. However, the substitution of a N atom for one of the O atoms in the Ti(OH)_4_ model corresponded to a nitrogen doping level of 25 atom %, which was much larger than the concentration of less than 0.2 atom % applied in the experiments.^87^ Therefore, the effects of nitrogen doping could be overestimated in the calculations results. A larger model capable of accurately simulating lower levels of doping would therefore be necessary for quantitatively accurate calculations.

#### 4.4.3 Ti 2p Core-Ionized States

Ti 2p orbital energies calculated using the Hartree–Fock method and the weighting coefficients estimated from these orbital energies are summarized in Table S9, and the total electronic state energies are shown in Table S10. The three orbital energies were determined to be approximately −18.08 hartree for Ti(OH)_4_ and −18.05 hartree for Ti(OH)_3_(NH_2_). Note that all of the −ε_2p_ values, when rounded up to the first decimal place, were 18.1 hartree. As a result, common weighting coefficients were adopted for both models. The calculation results for the Ti 2p core-ionized states are provided in Table 6. The calculated core-ionization energies derived from the three Ti 2p orbitals were averaged to obtain the *E*_2p_ value for each model and the resulting *E*_2p1/2_ and *E*_2p3/2_ values for Ti(OH)_3_(NH_2_) were found to be lower than those for Ti(OH)_4_. The results suggest that the Ti 2p peaks in the XPS spectrum should be shifted to lower energy due to nitrogen doping, in agreement with the experimental findings.^73,74^

Since the Ti 2p level was shifted upward as a consequence of the substitution of a N atom for an O atom (Table S9), the Ti 2p core-ionization energy was decreased. However, the calculated core-ionized energies of Ti(OH)_3_(NH_2_) were lower than those of Ti(OH)_4_ by 0.9–1.5 eV, and this shift was larger than the experimental values of 0.3–0.6 eV.^73,74^ This discrepancy can be attributed to the overestimated extent of nitrogen doping in the models, as with the discrepancy for the calculations of O 1s → LUMO core-excited states. The calculated core-ionization energies were decreased by approximately 15 eV following orbital optimization, suggesting that orbital relaxation had a significant effect.

Overall, the results of the VQD calculations suggest that nitrogen doping was responsible for the red-shift in the absorption band edge, the increase in the O 1s → Ti 3d excitation energy and the upward shift of the Ti 2p core-level, all of which were in qualitatively good agreement with the experimental data.

## 5 Conclusions

The VQD method has attracted attention as a promising quantum-classical hybrid algorithm for the calculation of excited states. In the present study, calculations of the core-excited states and core-ionized states for common molecules utilizing the VQD method were simulated on a classical computer, focusing on the penalty terms in the cost function. The weighting coefficients for these penalty terms were estimated on the basis of molecular orbital levels and the minimum deviations of the eigenvalues of the wave function regarding the spin and the number of electrons. Adopting this simple procedure allowed the core-level states to be successfully calculated. The relationship between the weighting coefficients and the resulting ansatz states was systematically examined on the basis of calculations of the O 1s core-ionized state for a water molecule. The results indicate that the weighting coefficients estimated by our procedure allowed the target states to be obtained by raising the cost of undesired states. The O 1s core-excited states and the Ti 2p core-ionized states for TiO_2_ and N-TiO_2_ photocatalysts serving as model compounds could also be calculated in the same manner as the valence excited states. The results of such calculations demonstrated that nitrogen doping induced a red-shift in the absorption band edge, increased the O 1s → Ti 3d excitation energy, and raised the Ti 2p core-level. These results were consistent with experimental findings, suggesting that VQD calculations can be applied to the analysis of functional materials in collaboration with experimental work. The results of this study are expected to provide valuable guidelines for future applications of the VQD method using quantum computers and also to inspire further development of the algorithm.

## Supporting Information Available

Schematic image of splitting of the 2p core-ionized doublet state (Figure S1); orbital energies and weighting coefficients used in the core-excited and core-ionized states of common molecules (Tables S1 and S2); Cartesian coordinates of the (TiO_2_)_16_ cluster (Table S3); Cartesian coordinates of the Ti(OH)_4_ and Ti(OH)_3_(NH_2_) cluster models (Table S4); orbital energies, weighting coefficients used in the VQD calculations, and total electronic state energies for the models of photocatalysts (Tables S5–S10) (PDF)
