# Supplementary material for: Calculation of Core-Excited and Core-Ionized States Using Variational Quantum Deflation Method and Applications to Photocatalyst Modeling
Source: ACS Omega. 2022 Mar 16;7(12):10840–53. doi: 10.1021/acsomega.2c01053 (PMC8973155; doi:10.1021/acsomega.2c01053)
Supplement: Supplementary file 1 — ao2c01053_si_001.pdf [file ao2c01053_si_001.pdf]

Supporting Information

Calculation of Core-Excited and Core-Ionized States Using Variational Quantum Deflation Method and Applications to Photocatalyst Modeling

Soichi Shirai,[†]* Takahiro Horiba[†] and Hirotoshi Hirai[†]

[†] Toyota Central R&D Labs., Inc., Nagakute, Aichi 480−1192, Japan

* Author to whom correspondence should be addressed.

E−mail: shirai@mosk.tytlabs.co.jp

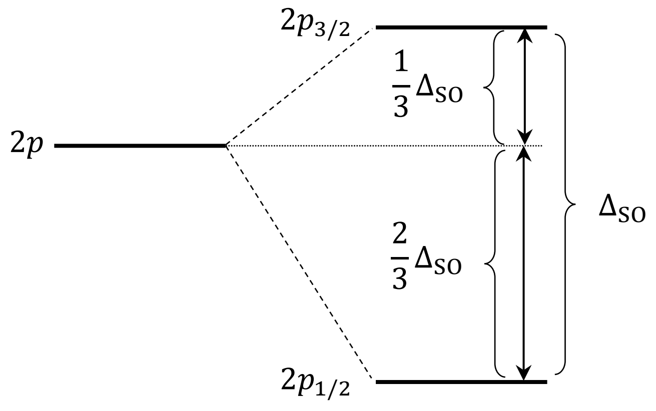

Figure S1. Splitting of the $2p$ core-ionized doublet state of an atom into $2p_{1/2}$ and $2p_{3/2}$ states due to spin-orbit interactions.

Table S1. Core 1s orbital energies (ε_{1s}), π* orbital energies ($\varepsilon_{\pi*}$) obtained using the Hartree-Fock method, their differences ($\varepsilon_{\pi*} - \varepsilon_{1s}$) and weighting coefficients in the cost function for the VQD calculations: overlap weights (β), s2 number weights (w_1), sz number weights (w_2) and particle number weights (w_3). All values are in hartrees. The $\varepsilon_{\pi*} - \varepsilon_{1s}$ value was rounded up to an integer prior to being used for the estimation of the weighting coefficients.

Molecule	Energy of core orbital		$\varepsilon_{\pi*}$	$\varepsilon_{\pi*} - \varepsilon_{1s}$	β	w_1	w_2	w_3
	Orbital	ε_{1s}						
CO	C 1s	−11.3612	0.1276	11.4888	12.0	21.4	48.0	12.0
	O 1s	−20.6649	0.1276	20.7925	21.0	37.4	84.0	21.0
H_2CO	C 1s	−11.3451	0.1356	11.4807	12.0	21.4	48.0	12.0
	O 1s	−20.5757	0.1356	20.7113	21.0	37.4	84.0	21.0
HCN	C 1s	−11.2919	0.1515	11.4434	12.0	21.4	48.0	12.0
	N 1s	−15.5989	0.1515	15.7504	16.0	28.5	64.0	16.0

Table S2. Core 1*s* orbital energies (ε_{1s}) obtained from Hartree-Fock calculations and weighting coefficients in the cost function for the VQD calculations: overlap weights (β), s2 number weights (w_1), sz number weights (w_2) and particle number weights (w_3). The ε_{1s} values were rounded up to the nearest integer when used for the estimation of weighting coefficients. All values are in hartrees.

Molecule	ε_{1s}	β	w_1	w_2	w_3
CH_4	−11.2152	12.0	21.4	48.0	12.0
NH_3	−15.5367	16.0	28.5	64.0	16.0
H_2O	−20.5508	21.0	37.4	84.0	21.0
FH	−26.2781	27.0	48.0	108.0	27.0

Table S3. Cartesian coordinates of the $(TiO_2)_{16}$ cluster for an optimized geometry. All coordinates are in Å.

Atom number	Atom	x	y	z
1	O	−2.743094	−4.930878	−1.644764
2	O	−3.223842	2.620283	−1.794587
3	TI	−1.491642	−3.525124	−1.651573
4	O	−2.349298	−2.078782	−1.656494
5	O	−0.446784	1.520713	−2.765294
6	TI	−1.351217	2.643908	−1.833069
7	O	−0.575438	4.286053	−1.952746
8	O	0.320667	−3.429621	−2.149929
9	TI	1.600307	−2.205244	−1.819556
10	O	1.428093	−0.742141	−2.786001
11	TI	1.506863	1.104128	−2.384429
12	O	1.734851	3.058321	−2.985297
13	TI	1.280138	4.393438	−2.035112
14	O	3.389917	−2.548544	−1.454051
15	O	3.352293	1.232853	−2.009813
16	O	2.706991	5.549568	−1.612746
17	TI	−3.297590	−4.721143	0.077714
18	O	−4.174841	−3.194449	0.205574
19	TI	−3.234491	−1.501819	0.249338
20	O	−3.991495	0.079553	−0.349306
21	TI	−3.502224	1.792519	−0.216095
22	O	−1.588480	−3.789473	0.254565
23	O	−1.365606	−1.161963	0.649103
24 [a]	O	−1.572274	1.808945	−0.062399
25	O	1.572274	−1.808945	0.062399
26	O	1.365606	1.161963	−0.649103
27	O	1.588480	3.789473	−0.254565
28	TI	3.502224	−1.792519	0.216095
29	O	3.991495	−0.079553	0.349306
30	TI	3.234491	1.501819	−0.249338
31	O	4.174841	3.194449	−0.205574
32	TI	3.297590	4.721143	−0.077714
33	O	−2.706991	−5.549568	1.612746
34	O	−3.352293	−1.232853	2.009813
35 [a]	O	−3.389917	2.548544	1.454051
36	TI	−1.280138	−4.393438	2.035112
37	O	−1.734851	−3.058321	2.985297
38	TI	−1.506863	−1.104128	2.384429
39 [a]	O	−1.428093	0.742141	2.786001
40 [a]	TI	−1.600307	2.205244	1.819556
41 [a]	O	−0.320667	3.429621	2.149929
42	O	0.575438	−4.286053	1.952746
43	TI	1.351217	−2.643908	1.833069
44	O	0.446784	−1.520713	2.765294
45	O	2.349298	2.078782	1.656494
46	TI	1.491642	3.525124	1.651573
47	O	3.223842	−2.620283	1.794587
48	O	2.743094	4.930878	1.644764

[a] Cut out from the $(TiO_2)_{16}$ cluster to prepare the $Ti(OH)_4$ model.

Table S4. Cartesian coordinates of the $Ti(OH)_4$ and $Ti(OH)_3(NH_2)$ cluster models for the optimized geometries. All coordinates are in Å.

Model	Atom number	Atom	x	y	z
$Ti(OH)_4$	1	TI	0.143200	0.068143	−0.098753
	2	O	−0.968933	−0.801362	1.207634
	3	O	−1.387677	−0.113054	−1.137972
	4	O	0.421414	1.757011	0.317694
	5	O	1.663469	−0.885107	−0.258325
	6	H	1.850555	−1.784273	−0.561348
	7	H	−0.531650	−1.073469	2.032125
	8	H	−0.249525	2.438470	0.472430
	9	H	−2.045952	−0.739775	−0.802893
$Ti(OH)_3(NH_2)$	1	TI	0.163467	0.043089	−0.101975
	2	N	−1.058976	−0.576716	1.247628
	3	O	−1.365352	−0.046302	−1.155998
	4	O	0.633124	1.717598	0.179693
	5	O	1.562620	−1.090119	−0.147826
	6	H	1.592837	−2.055431	−0.193166
	7	H	−0.782548	−0.775609	2.212358
	8	H	0.180257	2.471373	0.579572
	9	H	−1.781404	−0.906971	−1.313207
	10	H	−2.035728	−0.293731	1.217554

Table S5. The HOMO and LUMO energies obtained from Hartree-Fock calculations ($\varepsilon_{\mathrm{HOMO}}$ and $\varepsilon_{\mathrm{LUMO}}$), HOHO–LUMO gaps ($\varepsilon_{\mathrm{HOMO-LUMO}}$), and weighting coefficients for the cost function in the VQD calculations estimated from the $\varepsilon_{\mathrm{HOMO-LUMO}}$ values, comprising overlap weights (β), s2 number weights (w_1), sz number weights (w_2) and particle number weights (w_3). All values are in hartrees.

Model	$\varepsilon_{\mathrm{HOMO}}$	$\varepsilon_{\mathrm{LUMO}}$	$\varepsilon_{\mathrm{HOMO-LUMO}}$	Weighting coefficient			
				β	w_1	w_2	w_3
$Ti(OH)_4$	−0.4632	0.0463	0.5095	0.6	1.1	2.4	0.6
$Ti(OH)_3(NH_2)$	−0.3876	0.0517	0.4394	0.5	0.9	2.0	0.5

Table S6. S_0 and S_1 electronic state energies and HOMO → LUMO excitation energies calculated for the $Ti(OH)_4$ and $Ti(OH)_3(NH_2)$ models.

Model	Orbital	Electronic state energy (hartrees)		HOMO → LUMO excitation energy		
		S_0	S_1	(hartrees)	(eV)	(nm)
$Ti(OH)_4$	not optimized	−1150.3944	−1150.0545	0.3398	9.25	134.1
	optimized	−1150.3944	−1150.2764	0.1179	3.21	386.3
$Ti(OH)_3NH_2$	not optimized	−1130.5426	−1130.2608	0.2817	7.67	161.7
	optimized	−1130.5426	−1130.4352	0.1073	2.92	424.5

Table S7. O 1*s* orbital energies calculated using the Hartree-Fock method (ε_{1s}) and the weighting coefficients used in the VQD calculations. All values are in hartrees.

Model	ε_{1s}	$\varepsilon_{\mathrm{LUMO}}$	$\varepsilon_{1s-\mathrm{LUMO}}$	Weighting coefficient			
				β	w_1	w_2	w_3
$Ti(OH)_4$	−20.5447	0.0463	20.5910	20.7	36.8	82.8	20.7
	−20.5467		20.5930				
	−20.5772		20.6235				
	−20.5810		20.6273				
$Ti(OH)_3(NH_2)$	−20.5397	0.0517	20.5914	20.7	36.8	82.8	20.7
	−20.5624		20.6141				
	−20.5635		20.6152				

Table S8. Calculated electronic energies for the ground states (S_0) and O 1*s* → LUMO core-excited states, and the calculated O 1*s* → LUMO core-excitation energies.

Model	Orbital	S_0 (hartrees)[a]	O 1*s* → LUMO core-excited state (hartrees)[b]	Core-excitation energy	
				(hartrees)	(eV)
$Ti(OH)_4$	not optimized	−1150.3944	−1129.9646	20.4298	555.92
	optimized	−1150.3944	−1130.8285	19.5659	532.41
$Ti(OH)_3NH_2$	not optimized	−1130.5426	−1110.0964	20.4462	556.36
	optimized	−1130.5426	−1110.9545	19.5881	533.01

[a] Charge-neutral singlet ground state. [b] Averaged for each model.

Table S9. Ti 2p orbital energies calculated using the Hartree-Fock method (ε_{2p}) and the weighting coefficients used in the VQD calculations. All values are in hartrees.

Model	ε_{2p}			Weighting coefficient			
	Orbital 1	Orbital 2	Orbital 3	β	w_1	w_2	w_3
$Ti(OH)_4$	−18.0812	−18.0815	−18.0817	18.1	32.2	72.4	18.1
$Ti(OH)_3(NH_2)$	−18.0461	−18.0462	−18.0465	18.1	32.2	72.4	18.1

Table S10. Calculated electronic energies of the charge-neutral ground states (S_0) and Ti 2p core-ionized states, and core-ionization energies (E_{2p}).

Model	Orbital	S_0 (hartrees) [a]	Ti 2p core-ionized state (hartrees) [b]	E_{2p}	
				(hartrees)	(eV)
$Ti(OH)_4$	not optimized	−1150.3944	−1132.3129	18.0815	492.01
	optimized	−1150.3944	−1132.8492	18.0463	477.42
$Ti(OH)_3NH_2$	not optimized	−1130.5426	−1112.4963	17.5452	491.06
	optimized	−1130.5426	−1113.0502	17.4924	475.98

[a] Charge-neutral singlet ground state. [b] Averaged for each model.
